# Management of Traumatic Cerebral Venous Sinus Thrombosis: A United Kingdom and Ireland Survey on Practice Variation

**DOI:** 10.1089/neur.2023.0118

**Published:** 2024-06-06

**Authors:** Sheikh M. B. Momin, David J. Davies, Philip J. O’Halloran, Antonio Belli, Tonny Veenith, Ramesh Chelvarajah, on behalf of the Birmingham Traumatic Venous Sinus Thrombosis Consortium, Alex Leggate, David Bennett, Paul Brennan, Giles Critchley, Jack Wildman, Hadleigh Cuthbert, Laurence Glancz, Rebecca Chave-Cox, Daniel Odhiambo Ochieng

**Affiliations:** ^1^ Department of Neurosurgery, Queen Elizabeth Hospital, Birmingham, UK.; ^2^ Institute of Inflammation and Ageing, University of Birmingham, Birmingham, UK.; ^3^ Centre for Trauma Sciences Research, Institute of Inflammation and Ageing, University of Birmingham, Birmingham, UK.; ^4^ Department of Physiology & Medical Physics, Royal College of Surgeons of Ireland, University of Medicine and Health Sciences, Dublin, Ireland.; ^5^ Department of Intensive Care Medicine, Queen Elizabeth Hospital, Birmingham, UK.; ^6^ Centre for Human Brain Health, College of Life Sciences, University of Birmingham, Birmingham, UK.

**Keywords:** anticoagulation, survey, traumatic brain injury, venous sinus thrombosis

## Abstract

Traumatic cerebral venous sinus thrombosis (tCVST) is an increasingly recognized sequela of traumatic brain injury (TBI), with skull fractures and extradural hematomas overlying venous sinuses recognized as risk factors. Although it may be treated with anticoagulation, the decision to treat tCVST is nuanced by the risk of new or worsening hemorrhage. Presently, there are no guidelines on the investigation and management of tCVST. Therefore, we conducted a UK- and Ireland-wide practice variation survey. A 17-question survey was sent via Google Forms to neurosurgeons and intensive care doctors of at least ST3 (registrar) level and above in the UK and Ireland and distributed by the Society of British Neurological Surgeons and investigators of the Sugar or Salt trial between May 9, 2023, and September 15, 2023. Data were extracted from the survey for both qualitative and quantitative analyses. There were 41 respondents to the survey, 18 (43.9%) of whom were consultant neurosurgeons. Fifty-four percent of the respondents performed a computed tomography intracranial venogram to investigate for tCVST where there was a skull fracture overlying or adjacent to a venous sinus, whereas 43.9% performed these at the time of TBI diagnosis. Around three-fourth of the respondents anticoagulate for tCVST, largely within 3 days post-TBI. A range of hemorrhagic and thrombotic complications have been observed following decisions to treat and withhold treatment of tCVST, respectively. Around two-third of the respondents conducted follow-up imaging in confirmed tCVST. None of the respondents had an established departmental protocol for the management of tCVST. This UK- and Ireland-wide survey on the management of tCVST revealed a variation in its diagnosis, treatment, and follow-up with no departmental protocol established. The optimal diagnostic pathway, management protocol, and follow-up of patients with tCVST remain unknown and should be the subject of future studies.

## Introduction

Traumatic cerebral venous sinus thrombosis (tCVST) refers to clot formation in one of the dural venous sinuses secondary to traumatic brain injury (TBI). Compared with spontaneous cerebral venous sinus thrombosis (CVST), which tends to occur in younger patients with a predilection to venous thromboembolism, tCVST is usually caused by direct mechanical impact to the sinus wall,^1^ with penetrating injuries, skull fractures, and extradural hematomas overlying the venous sinus recognized as risk factors.^2–4^ A meta-analysis of 6861 patients with TBI quantified a pooled incidence of tCVST of 4%, increasing to 26.2% in patients with skull fractures adjacent to venous sinuses.^2^

tCVST is increasingly recognized as an important sequela of severe TBI, associated with increased mortality^1^ as well as complications; these include veno-occlusive complications also present in spontaneous CVST: progression to cerebral oedema, venous infarction, and hemorrhage in the drainage territory of the thrombosed venous sinus.^5^ Moreover, there is a risk of refractory intracranial hypertension and disruption to cerebrospinal fluid drainage pathways. However, the decision to treat tCVST with anticoagulation is complicated by the frequent association with other traumatic intracranial hemorrhage, which may worsen with anticoagulation therapy. A retrospective cohort study of 137 patients with tCVST associated with skull fractures found a significantly lower in-hospital mortality in 82 patients treated with unfractionated heparin following >72 h of stable intracranial traumatic hemorrhages compared with 55 patients who were managed conservatively. This is despite seven patients having new or worsening hemorrhages (with one death from this group).^6^ In another retrospective cohort of 38 patients with tCVST, 22 of whom were treated with anticoagulation, six patients had hemorrhagic complications (three extracranial and three intracranial—leading to one mortality).^7^ However, a UK series showed no difference in outcome with or without anticoagulation.^8^ There is no consistent protocol in the initiation timing and dosing of anticoagulation (if given) in tCVST.^1^ Similarly, there is no consensus in the imaging modalities used to diagnose tCVST and when they are indicated. Computed tomography (CT) venography has been preferentially used in many studies of adult tCVST, with magnetic resonance (MR) venography preferred in pediatrics to avoid ionizing radiation.

Moreover, guidelines on venous sinus thrombosis recommend treatment with anticoagulation but do not specifically consider traumatic etiology.^9–11^ Anecdotally in our institution, we also noted variability in the investigation and management of tCVST. To date, there are no UK- and Ireland-wide evaluation of management practice. Therefore, we conducted a UK- and Ireland-wide survey on practice variation in the management of tCVST.

## Methods

A 17-item survey (Supplemental Data S1) was created via Google Forms and distributed online to neurosurgeons and intensive care doctors of at least PGY3-equivalent (registrar) or above via the Society of British Neurological Surgeons (SBNS), British Neurosurgical Trainees’ Association, and the Salt or Sugar [SOS (warwick.ac.uk)] trial investigator mailing lists after being ratified by the SBNS Academic Committee. The SOS trial is an ongoing study investigating whether hypertonic saline or mannitol in TBI improves neurological function clinically and cost-effectively at 6 months. Investigators of this trial consisted of neurointensive care and neurosurgery consultants and registrars throughout the UK who we felt would provide an appropriate neurointensive care perspective to the survey. Survey questions included demographic information about respondents, as well as questions about the following topics:

1. Indication and timing of CT intracranial venogram to diagnose tCVST (Q3 and Q4).

Within this section, common indications to investigate tCVST, such as skull fracture overlying a sinus or anatomical blood distribution,^2,4^ were included.

Use of anticoagulation to treat tCVST (Q5–Q8)

Respondents indicated whether they used anticoagulation to treat tCVST, with further questions regarding timing and dosing of anticoagulation were posed.

Complications and follow-up for tCVST (Q9–Q15)

Questions about hemorrhagic (where anticoagulation was used) and thrombotic (where anticoagulation was not used) complications resulting from the management of tCVST were posed, as well as the radiological follow-up of tCVST.

Respondents were included as collaborative authors in this article as part of the Birmingham Traumatic Venous Sinus Thrombosis Group. Respondents were also asked if they were interested in participating in a prospective study on the management of tCVST. The survey was open for responses from May 9, 2023, to August 12, 2023. Data were collected and handled in accordance with the Information Commissioner's Office and the Data Protection Act 2018, while participant consent was sought and obtained at the beginning of the survey.

## Results

There were 41 responses to the survey during the study period.

### Demographics

**FIG. 1.**
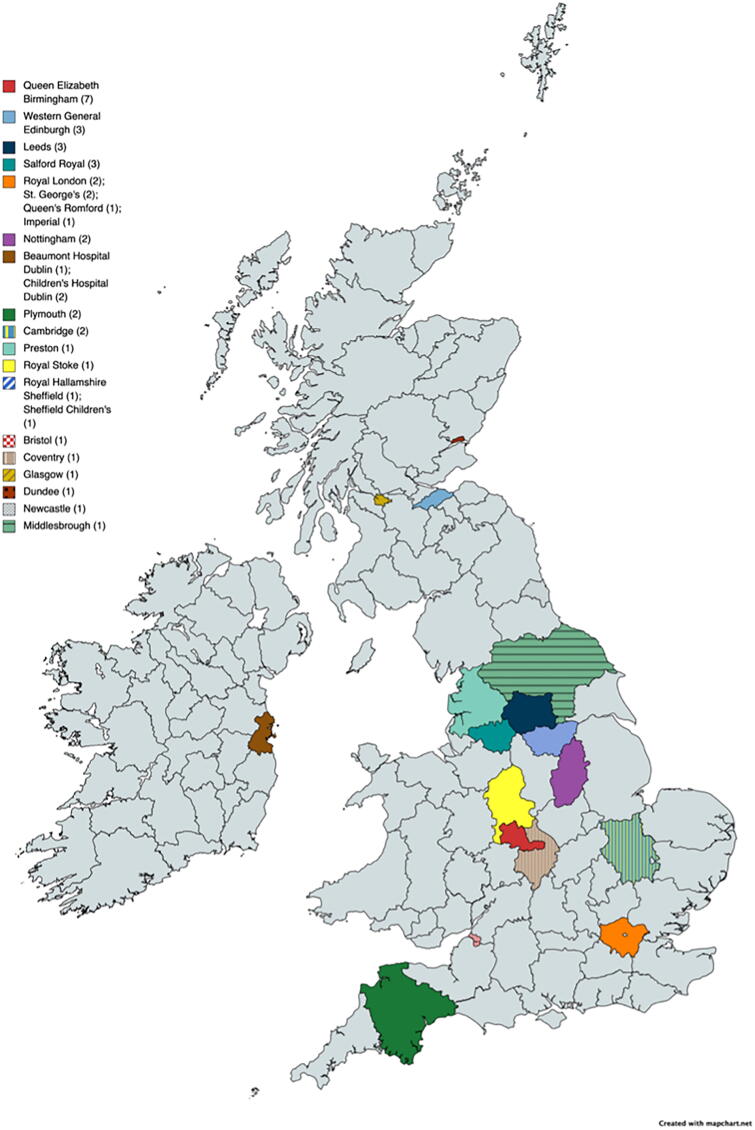
Geographical distribution of survey respondents.

Responses were received from 23 UK and Ireland neurosurgical units, with the Queen Elizabeth Hospital Birmingham having the most respondents (17.1%), as shown in Figure 1.

Seventy-five percent of the respondents were neurosurgeons, with 43.9% of them being consultant neurosurgeons.

### Investigation of tCVST

**FIG. 2.**
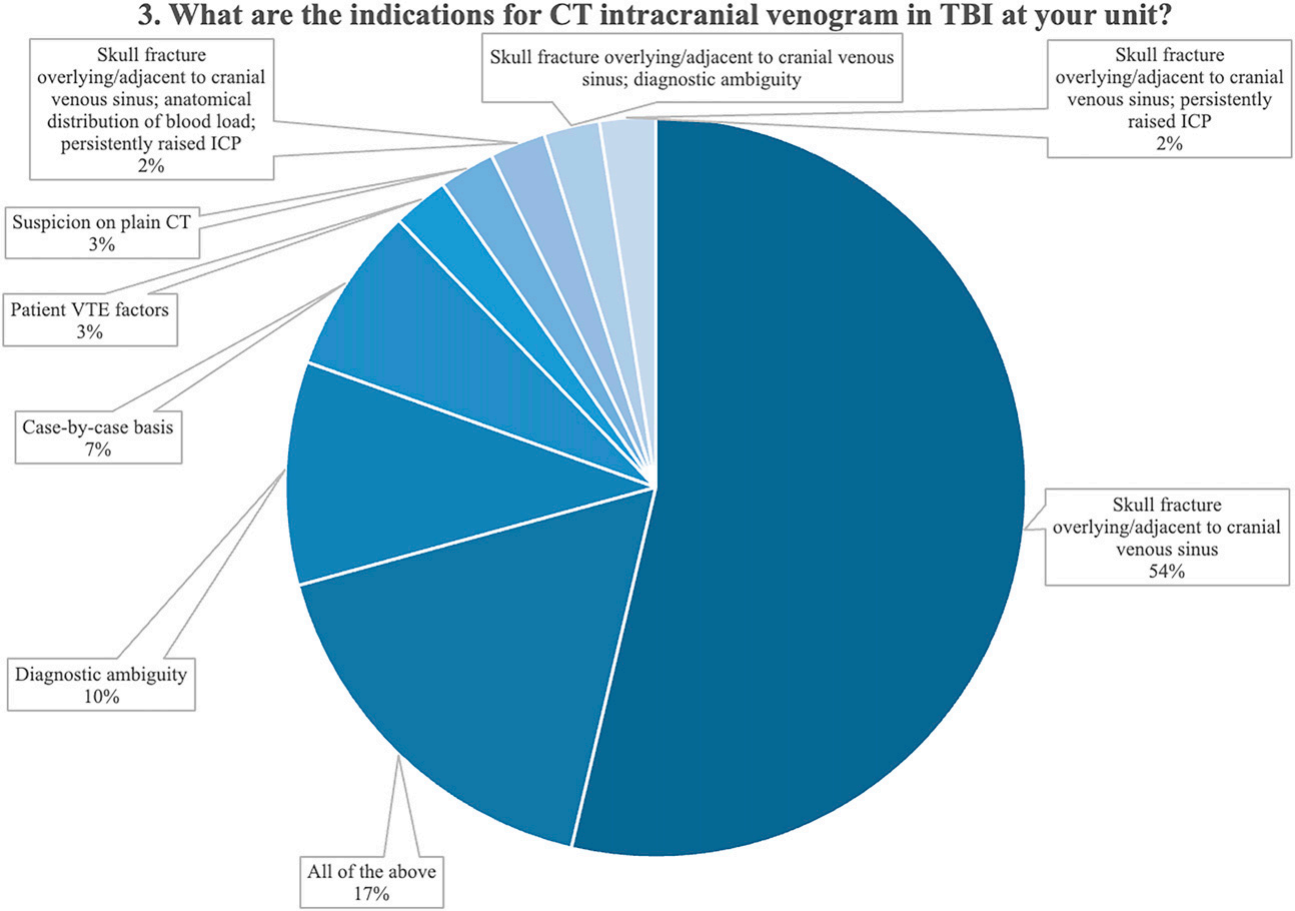
Indications for CT intracranial venogram to investigate for suspected tCVST. CT, computed tomography; tCVST, traumatic cerebral venous sinus thrombosis.

Two questions were related to the initial investigation of tCVST (Q3 and Q4). There were five predefined responses to Question 3: “What are the indications for CT intracranial venogram in TBI at your unit?” (Supplemental Data S1). A slight majority of respondents (54%) indicated that they would perform a CT intracranial venogram to investigate tCVST if there was a skull fracture overlying or adjacent to a venous sinus (Fig. 2). A further 17% (seven) of the respondents indicated that they would investigate for tCVST for all the indications listed in the questionnaire (skull fracture overlying or adjacent to a venous sinus, anatomical distribution of blood load, persistently raised intracranial pressure (ICP), diagnostic ambiguity, and patient venous thromboembolism (VTE) factors).

**FIG. 3.**
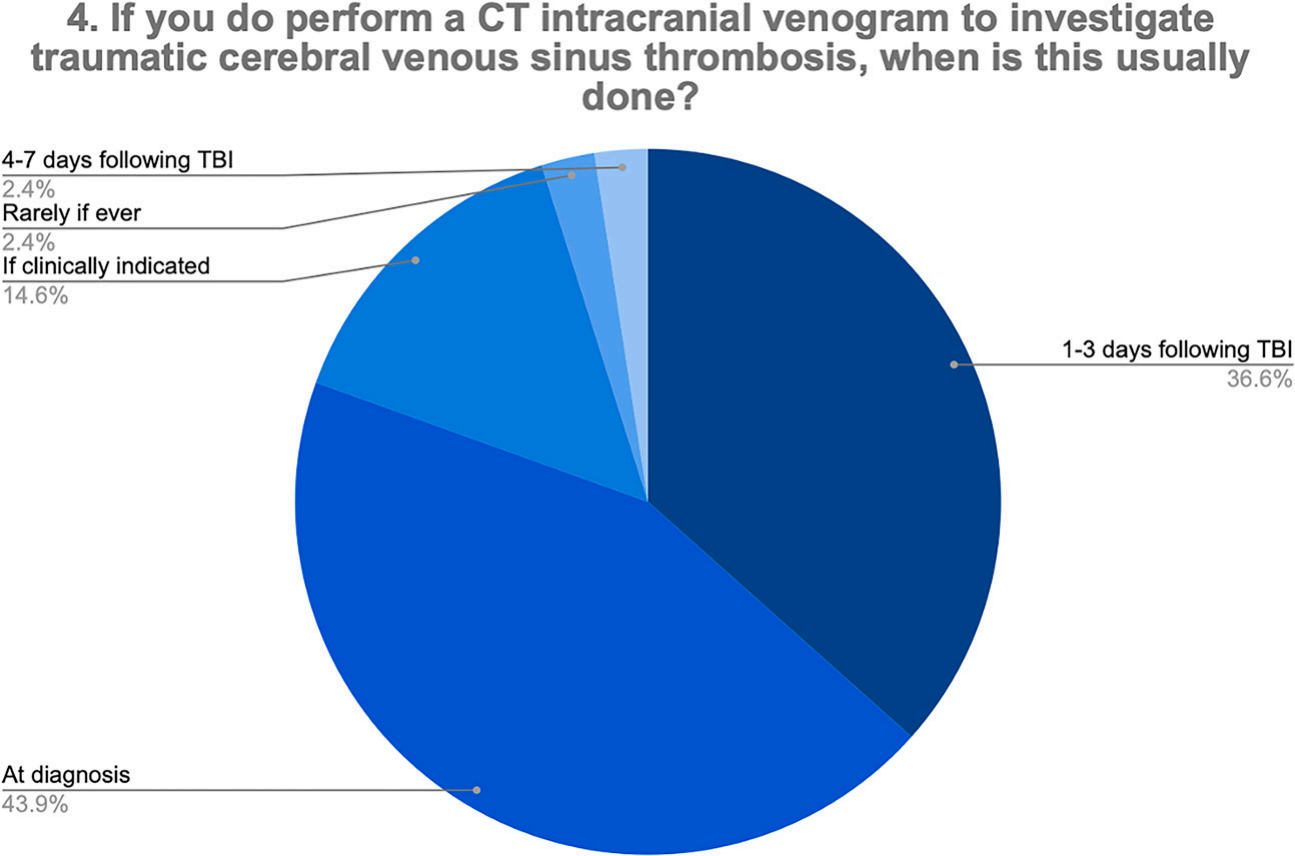
Timing of CT intracranial venogram to investigate tCVST. CT, computed tomography; tCVST, traumatic cerebral venous sinus thrombosis.

In addition, 43.9% of the respondents indicated that they would perform a CT venogram to diagnose tCVST at TBI diagnosis, whereas 36.6% indicated that they would perform this at 1–3 days post-injury (Fig. 3). One respondent rarely investigated for tCVST.

### Treatment of tCVST

Four questions (Q5–Q8) related to treatment strategy for tCVST.

**FIG. 4.**
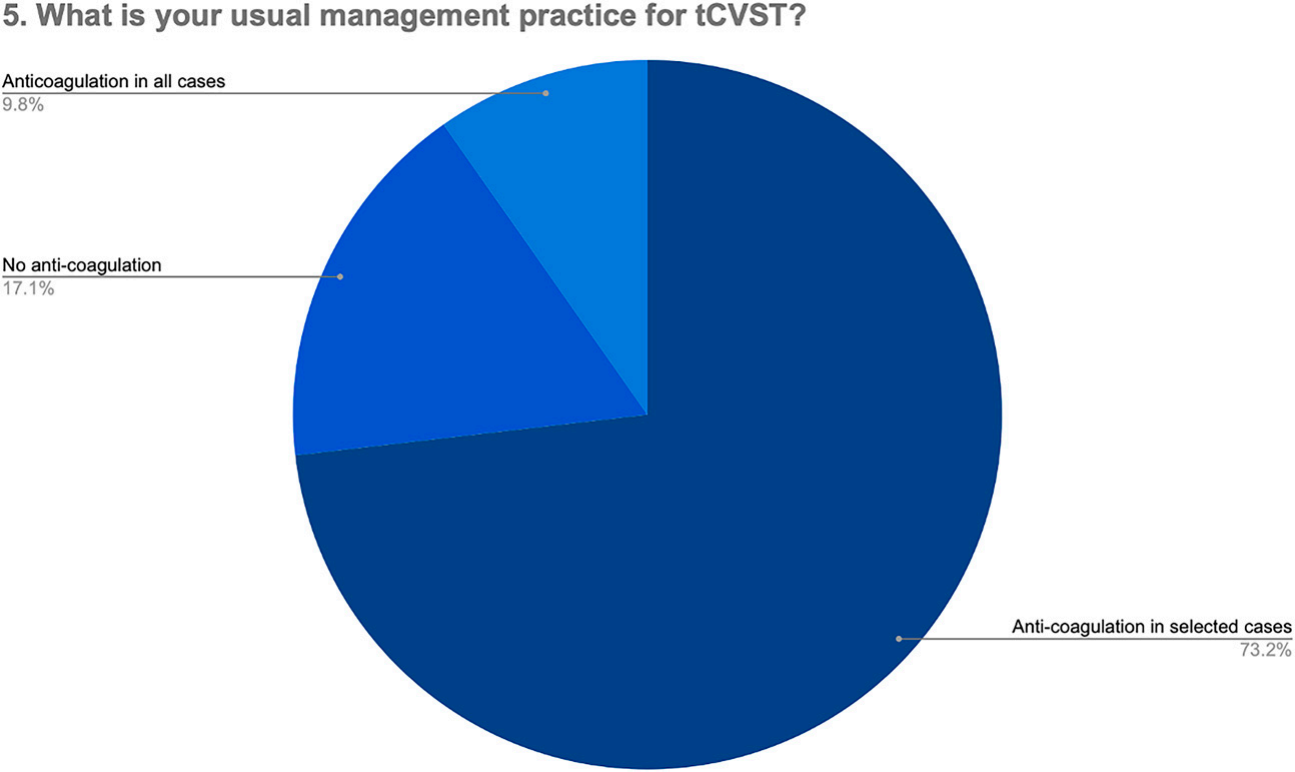
Management practice for confirmed tCVST. tCVST, traumatic cerebral venous sinus thrombosis.

In relation to Q5 (“What is your usual management practice for tCVST?”), only 9.8% of the respondents indicated that they would not give anticoagulation at all for tCVST, whereas around three-fourth of the respondents indicated that they would give anticoagulation in selected cases (Fig. 4).

A variety of free-text responses were given on criteria for starting anticoagulation for tCVST (Q6; Supplemental Data S2). The most common themes given were progressive or occlusive VST, likelihood of surgical intervention, and uncontrolled ICP. Several respondents commented that they would make these decisions alongside the local hematology/neurology/stroke teams.

**FIG. 5.**
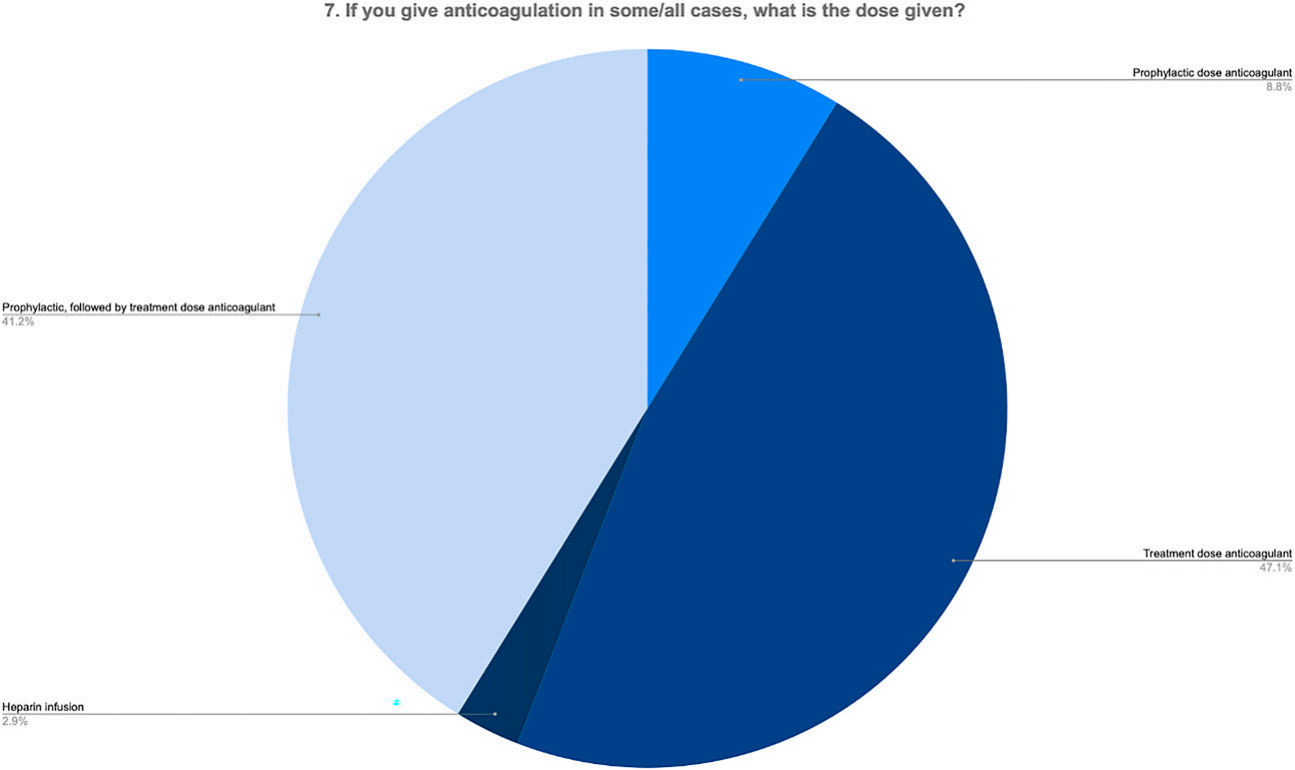
Anticoagulation dosing strategy for confirmed tCVST. tCVST, traumatic cerebral venous sinus thrombosis.

When asked about dosing of anticoagulation in treated tCVST (Q7), most respondents managed tCVST with treatment-dose anticoagulation, with 47.1% giving this at diagnosis, whereas 41.2% started with prophylactic, followed by treatment-dose anticoagulation (Fig. 5).

**FIG. 6.**
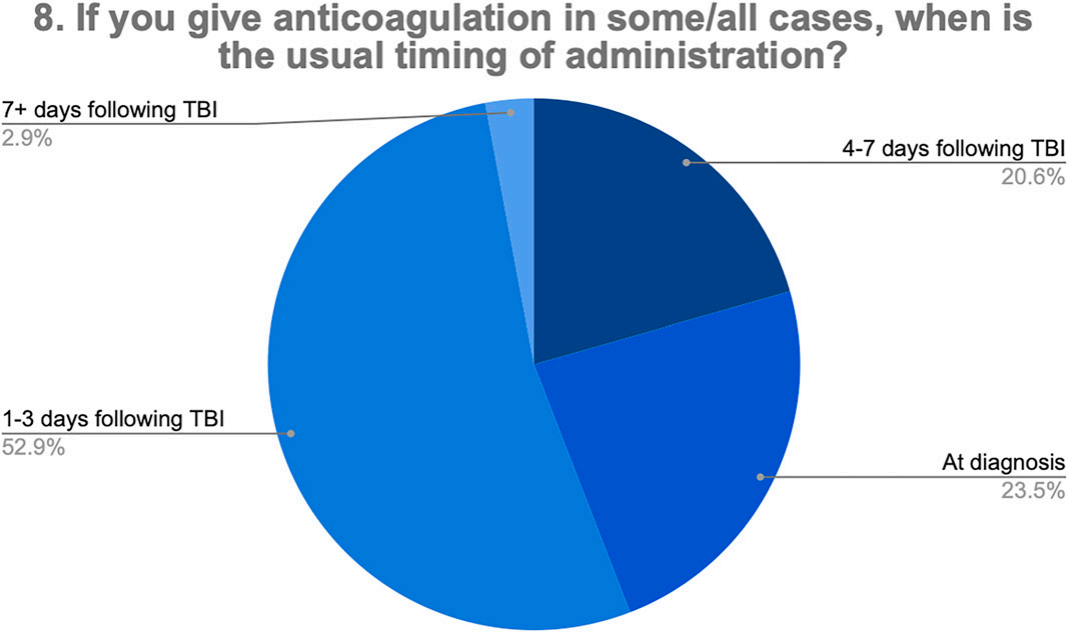
Timing of anticoagulation for confirmed tCVST. tCVST, traumatic cerebral venous sinus thrombosis.

When respondents who gave anticoagulation were asked about timing of anticoagulation (Q8), the majority (52.9%) started anticoagulation 1–3 days following TBI, whereas one respondent gave anticoagulation 7+ days after TBI (Fig. 6).

### Complications of managing tCVST

Four questions (Q9–Q12) related to the presence of complications from treating tCVST with anticoagulation, as well as withholding anticoagulation. Three predefined complications from treating and withholding treatment for tCVST were provided (Q9 and Q11) with an option for respondents to include other observed complications.

#### Complications from treating tCVST

**FIG. 7.**
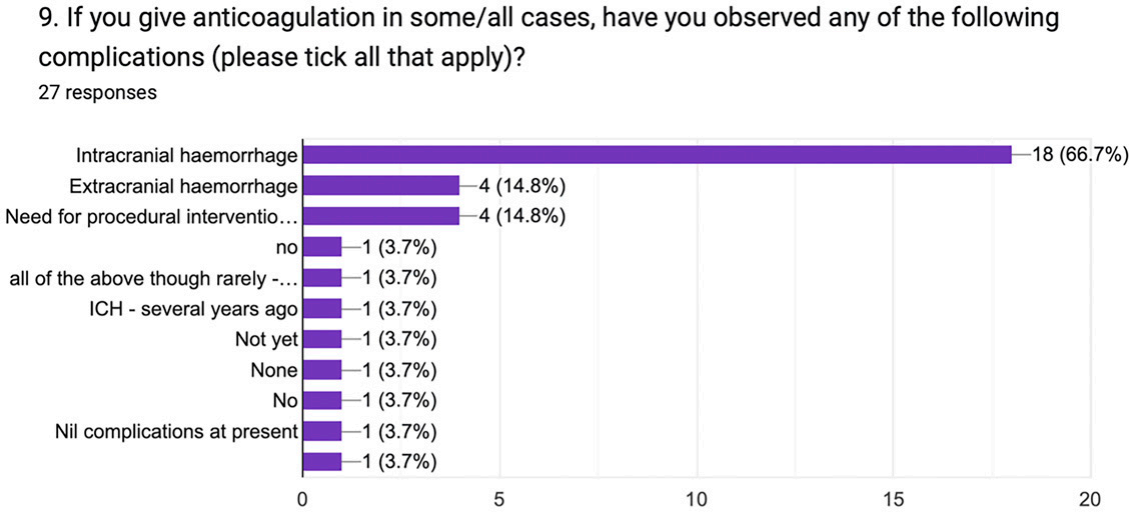
Observed complications following anticoagulation administration for tCVST. tCVST, traumatic cerebral venous sinus thrombosis.

It was found that 66.7% of the respondents giving anticoagulation observed intracranial hemorrhage in concurrence, with four respondents each observing extracranial hemorrhage and the need for procedural intervention (e.g., evacuation of intracerebral hematoma) following anticoagulation administration (Fig. 7).

**FIG. 8.**
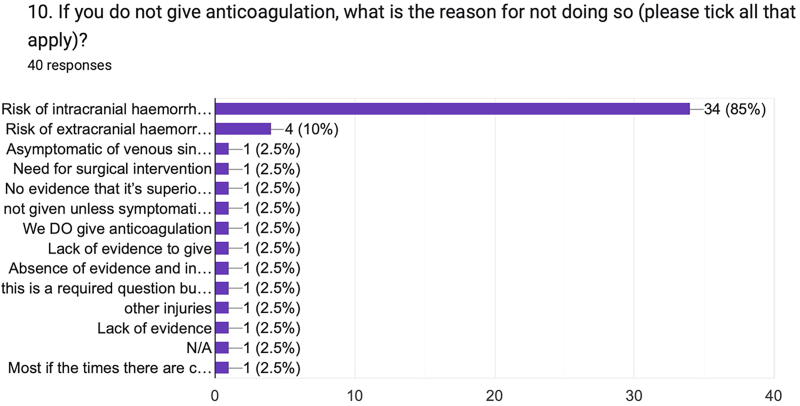
Reasons given for withholding anticoagulation in tCVST. tCVST, traumatic cerebral venous sinus thrombosis.

As expected, most respondents who did not give anticoagulation for tCVST (Q10) were concerned about the risk of intracerebral hemorrhage (85%), highlighting the common dilemma faced by clinicians in managing tCVST (Fig. 8). Four respondents (10%) stated they did not give anticoagulation because of lack of evidence of efficacy of this treatment.

#### Complications from withholding treatment for tCVST

**FIG. 9.**
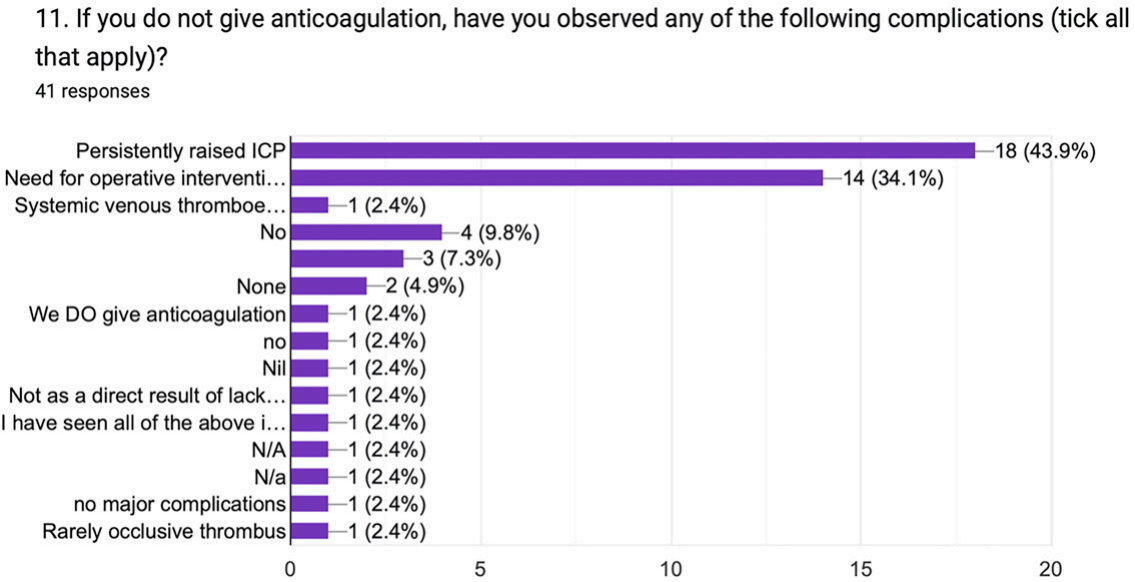
Observed complications following withholding anticoagulation in tCVST. tCVST, traumatic cerebral venous sinus thrombosis.

Where anticoagulation was not given (Q11), 43.9% of the respondents observed persistently raised ICP, and 34.1% observed a need for operative intervention (Fig. 9). Twelve respondents (29%) specifically reported no complications following withholding anticoagulation. One respondent noted that “I have seen all of the above [predefined complications] in the absence of anticoagulation as well as when it is given.”

Interestingly, about an even split of respondents felt it was more important to avoid hemorrhagic complications by anticoagulating a patient with tCVST (Q12) compared with a venous infarctive stroke in an un-anticoagulated patient with tCVST.

### Follow-up in tCVST

Two questions related to follow-up strategy in tCVST (Q13 and Q14). Around two-third of respondents conduct follow-up imaging for patients with tCVST (Q13).

**FIG. 10.**
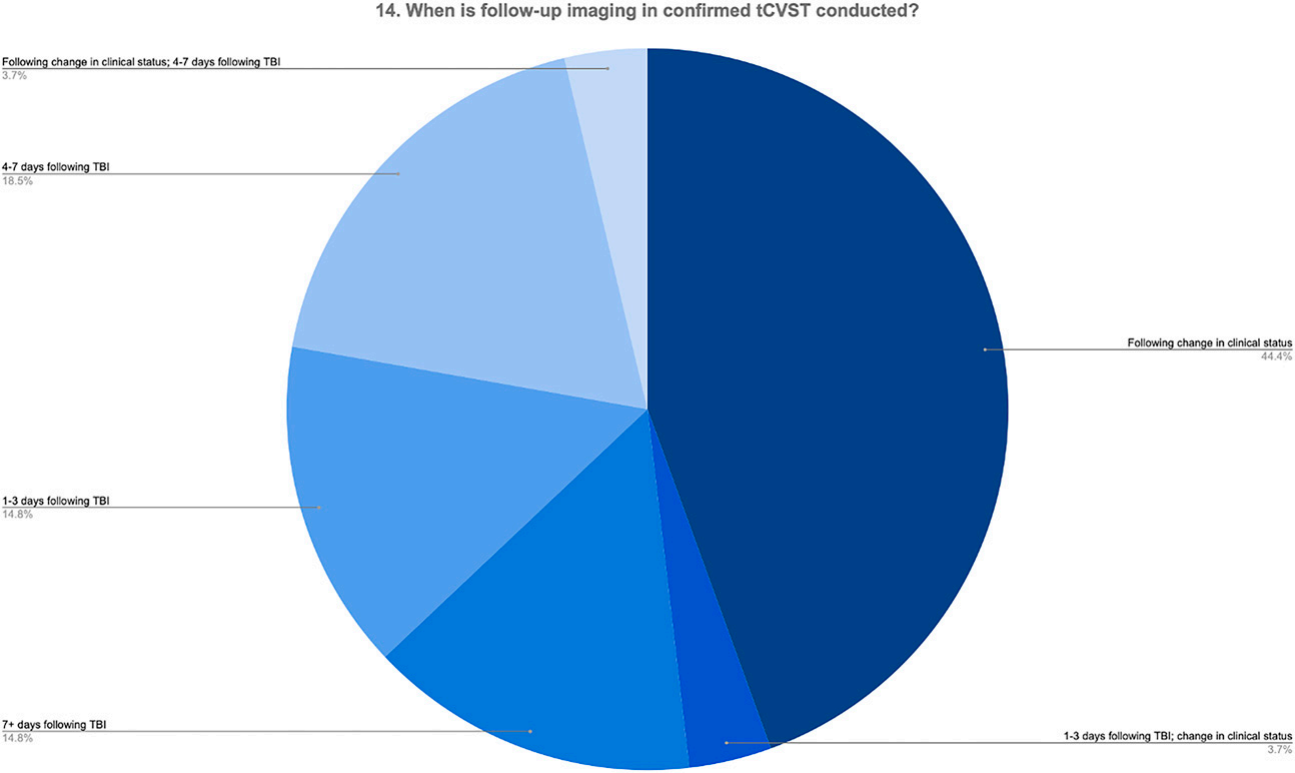
Timing of follow-up imaging in patients with confirmed tCVST. tCVST, traumatic cerebral venous sinus thrombosis.

Most respondents (40.7%) conducted this imaging following a change in clinical status (e.g., increasing ICP or if neurological status was not improving), whereas a minority of respondents conducted this 1–3, 4–7, or 7+ days following TBI. This is shown in Figure 10.

None of the respondents worked in a department with an established protocol for the management of tCVST, while just over half of the respondents (52.5%) were interested in participating in a prospective multicenter observational study on the use of anticoagulation for tCVST.

## Discussion

This qualitative national practice survey demonstrates considerable practice variation in the initial diagnosis, treatment, and follow-up of tCVST. Consistent with the literature,^1,2^ most respondents conducted CT intracranial venogram in the presence of a skull fracture overlying or adjacent to a venous sinus. Most respondents in our survey favored early CT venogram to diagnose tCVST (73% within 3 days), which is reflected in other studies on tCVST,^2,8^ although six respondents (15%) indicated that they would do so on clinical suspicion rather than a specific timeframe.

There is insufficient high-quality evidence on the optimal dosing and timing of anticoagulation, as well as outcomes following different anticoagulation strategies (e.g., low-molecular-weight heparin or unfractionated heparin).^1^ In our survey, most respondents favored a more aggressive approach, with around three-fourth of the respondents anticoagulating for tCVST and nine-tenth using therapeutic anticoagulation; there was a roughly even split on whether to give treatment dose or prophylactic dose at the outset. However, most respondents reported administering treatment-dose anticoagulation by 72 h post-injury. This is a complex decision dependent on multiple injury- and patient-related factors. For instance, in a published series of four patients from our unit with tCVST,^12^ three were treated with anticoagulation at 1, 4, and 23 days post-injury. The patient anticoagulated day 1 post-injury was noted to have refractory intracranial hypertension despite maximal medical management and cerebrospinal fluid (CSF) diversion. Propagation of the thrombus was found on serial CT venography, and a heparin infusion was commenced to manage ICP. Despite this, ICP remained raised, and despite a subsequent posterior fossa decompression, the patient deteriorated and eventually died. In contrast, the patient anticoagulated at 4 days post-TBI had a traumatic hemopneumothorax and rib fracture requiring fixation, with repeat CT venogram not demonstrating propagation; he eventually improved to GCS 15 and discharged to a rehabilitation facility. The patient anticoagulated at day 23 had delayed anticoagulation due to traumatic contusions and subdural hematoma, whereas the un-anticoagulated patient was E3V3M6 at presentation with an extradural hematoma overlying a left transverse sinus thrombus; both patients had no propagation of thrombus on CT venogram and a good neurological outcome.

These cases highlight the challenges in decision making on anticoagulating patients with tCVST. This was reflected in the free-text comments from respondents on their criteria for anticoagulating in tCVST (Q6; Supplementary Data S1), with response themes centering on the extent of traumatic intracranial hemorrhage, need for potential surgical intervention, and whether the thrombus was occlusive or whether ICP was refractory. Six respondents stated that they consult with their respective hematology and/or neurology teams, specialties with greater familiarity in managing CVST, on this decision making. Moreover, there was a roughly even split between respondents who felt it was important to treat therapeutically to minimize complications of tCVST as those who felt it important to withhold therapeutic anticoagulation to prevent hemorrhagic consequences (Q12).

Indeed, tCVST presents an alternative clinical problem compared with spontaneous CVST with respect to the presence of concomitant traumatic intracranial hemorrhage and extracranial injury, as well as presenting neurological status. These factors are not captured in current management guidelines.^10,11^ Survey responses reflect the hemorrhagic and thrombotic complications arising from decisions to treat or withhold treatment for tCVST. Two-third of the respondents had observed worsening intracranial hemorrhage following anticoagulation, whereas 43.9% had noted persistently raised ICP when anticoagulation was withheld; interestingly, more respondents noted that operative intervention was required after anticoagulation was withheld compared with when it was given (14 vs. 4 respondents). Nonetheless, a largely even split of respondents thought that it was important to avoid hemorrhagic complications on account of anticoagulating patients with tCVST, as those respondents felt the need to minimize the risk of a venous infarct by anticoagulating (Q12). This likely reflects the professional background of the survey respondents (neurosurgeons and intensive care doctors), and it would be interesting to note responses to this question from other professionals who manage this condition (e.g., neurologists and hematologists).

Hemorrhagic and thrombotic complications have been reported following tCVST. The rate of venous infarction following tCVST is reportedly 7–18%,^13–15^ with 11% developing CVST-associated intracerebral hematoma in one series.^14^ Severe intracranial hemorrhage following anticoagulation was reportedly 9–14% in two studies,^6,7^ with one death reported in each study. However, in one cohort study, there was a lower in-hospital mortality in the anticoagulated group despite the hemorrhagic complications, with anticoagulation found to be a negative predictor of in-hospital mortality in logistic regression analysis.^6^

Given the negative effect of tCVST on ICP in acute severe TBI, the most common practice for respondents was unsurprisingly to conduct follow-up imaging following a change in clinical status (e.g., refractory intracranial hypertension or lack of neurological improvement) rather than a fixed timepoint following initial tCVST diagnosis. There is no high-quality evidence in the literature on whether and when follow-up imaging should be conducted to assess, for instance, for recanalization/progression of thrombus following decisions to give or withhold anticoagulation^1^; recanalization rates are reported between 23% and 100%.^6–8,16^

Long-term outcomes in tCVST with persistent occlusion are also unknown.

Our survey has several important limitations. First, despite a relatively lengthy data collection period, we achieved only 41 responses, a fraction of the estimated UK and Ireland neurosurgical/intensive care community. The online survey format distributed by email, the optional nature of the survey, and survey fatigue are likely all contributing factors. Moreover, 14/41 respondents worked in neurosurgical units with a pediatric service, with three working in purely pediatric neurosurgical units. The questionnaire did not differentiate the management of pediatric tCVST; hence, conclusions about UK- and Ireland-based practice in this group cannot be drawn. The literature suggests that the approach in pediatric cases differs with the use of MR venography as the preferred diagnostic modality to avoid ionizing radiation,^1^ with recanalization rates between 70% and 86%. Despite these limitations, the number of respondents is comparable with that of other recent UK-based neurosurgical practice variation surveys,^17–19^ and there is a reasonable geographic spread of responses.

Second, the findings of the survey provide level 5 evidence^20^ on the management of tCVST, clearly reflecting anecdotal experiences and individual opinions rather than objective controlled data. This is particularly reflected in Q10 and Q11, where respondents reported a high observed proportion of hemorrhagic complications in tCVST. Nevertheless, as an entity without clear guidelines on investigation, treatment, and follow-up, we consider the survey format to be a useful starting point to understand current UK and Ireland management strategy.

Several studies have identified the need for prospective studies on tCVST to improve the evidence for investigation, treatment, and follow-up.^1,2,6,8^ In total, 21 survey respondents (51.2%) indicated they would be interested in participating in a prospective multicenter observational study on the use of anticoagulation for tCVST, with 13 respondents (31.7%) “maybe” interested in such a study. Therefore, there is possibly an appetite in the UK and Ireland neurosurgical/intensive care community to undertake such a study, which we have proposed leading within the umbrella of the “Birmingham Traumatic Venous Sinus Thrombosis Group.” Moreover, an upcoming UK-based randomized control trial on timing of VTE prophylaxis following TBI (TOP-TBI) may also provide further evidence in the tCVST population.

In conclusion, we have collated the views of UK- and Ireland-based neurosurgeons and intensive care doctors on the management of tCVST. Survey respondents favored early investigation of suspected tCVST (<3 days), particularly in patients with skull fracture across venous sinuses, with a preference to treat tCVST <3 days following TBI, with follow-up imaging conducted following changes in clinical status. This may serve as a baseline for further prospective studies on the management of tCVST.

## Birmingham Traumatic Venous Sinus Thrombosis Consortium

Alex Leggate, Department of Neurosurgery, Queen’s Medical Centre, Nottingham, UK; David Bennett – Department of Neurosurgery, Ninewells Hospital and Medical School, Dundee, UK; Paul Brennan – Department of Neurosurgery, Western General Hospital, Edinburgh, UK; Giles Critchley, Department of Neurosurgery, Royal Sussex County Hospital, Brighton, UK; Jack Wildman, Department of Neurosurgery, Southmead Hospital, Bristol, UK; Hadleigh Cuthbert, Department of Paediatric Neurosurgery, Birmingham Children’s Hospital, Birmingham, UK; Laurence Glancz, Department of Neurosurgrery, Queen’s Medical Centre, Nottingham, UK; Rebecca Chave-Cox, Department of Neurosurgery, Leeds General Infirmary, Leeds, UK; Daniel Odhiambo Ochieng, Department of Paediatric Neurosurgery, Sheffield Children’s Hospital, Sheffield, UK.

## Abbreviations Used

CSF: cerebrospinal fluid
CT: computed tomography
CVST: cerebral venous sinus thrombosis
ICP: intracranial pressure
MR: magnetic resonance
TBI: traumatic brain injury
VTE: venous thromboembolism
