# Supplementary material for: Management of Traumatic Cerebral Venous Sinus Thrombosis: A United Kingdom and Ireland Survey on Practice Variation
Source: Neurotrauma Rep. 2024 Jun 6;5(1):540–51. doi: 10.1089/neur.2023.0118 (PMC11285999; doi:10.1089/neur.2023.0118)
Supplement: Supplementary Data S1 [file neur.2023.0118_supplementarymaterial_1.docx]

**Supplementary Material 1: Survey Questionnaire**

**National practice variation survey on the diagnosis and management of traumatic cerebral venous sinus thrombosis**

1. Which neurosurgical unit do you work in?
2. What is your grade?
   1. Consultant Neurosurgeon
   2. Neurosurgery Clinical Fellow
   3. Neurosurgery Trainee
   4. Intensive Care Consultant
   5. Intensive Care Clinical Fellow
   6. Intensive Care Trainee
   7. Other (please specify)
3. What are the indications for CT intracranial venogram at your unit? Please tick all that apply
   1. Skull fracture going through cranial venous sinus
   2. Diagnostic ambiguity – e.g. bleeding pattern, but history not suggestive of venous sinus thrombosis
   3. Persistently raised ICP
   4. Anatomical distribution of blood load
   5. Patient VTE risk factors
   6. Other (please specify)
4. If you do perform a CT intracranial venogram, when is this usually done?
   1. At diagnosis
   2. 1-3 days following TBI
   3. 4-7 days following TBI
   4. 7+ days following TBI
   5. Other
5. What is your usual management practice for traumatic venous sinus thrombosis?
   1. Anticoagulation in all cases
   2. Anticoagulation in selected cases (please elaborate below)
   3. No anticoagulation
6. If you give anticoagulation in selected cases, what are your criteria?

**[Free text]**

1. If you give anticoagulation in some/all cases, what is the dose given?
   1. Treatment dose anticoagulant
   2. Prophylactic dose anticoagulant
   3. Prophylactic, followed by treatment dose anticoagulant
   4. Other
2. If you give anticoagulation in some/all cases, when is the timing of administration?
   1. At diagnosis
   2. 1-3 days following TBI
   3. 4-7 days following TBI
   4. 7+ days following TBI
3. If you give anticoagulation in some/all cases, have you observed any of the following complications?
   1. Intracranial haemorrhage
   2. Extracranial haemorrhage
   3. Need for procedural intervention for haemorrhage
   4. Other
4. If you do not give anticoagulation, what is the reason for not doing so?
   1. Risk of intracranial haemorrhage
   2. Risk of extracranial haemorrhage
   3. Other
5. If you do not give anticoagulation, have you observed any of the following complications?
   1. Persistently raised ICP
   2. Need for operative intervention (e.g. decompressive craniectomy
   3. Systemic venous thromboembolism
   4. Other
6. In your opinion which do you think is more important to avoid:
   1. a venous infarctive stroke in an un-anticoagulated patient with tCVST
   2. haemorrhagic complications in an anticoagulated patient with tCVST
7. Do you conduct follow-up imaging in patients with traumatic venous sinus thrombosis?
   1. Yes
   2. No
8. If yes, when is this conducted?
   1. 1-3 days following TBI
   2. 4-7 days following TBI
   3. 7+ days following TBI
   4. Following change in clinical status - e.g. increasing ICP, neurological status not improving

**[Free text]**

1. Does your unit have an established protocol for the management of traumatic venous sinus thrombosis?
   1. Yes
   2. No
2. If ‘Yes’, please elaborate further:

**[free text]**

1. Would you be interested in participating in a prospective multi-centre observational study on the use of anticoagulation for the management of traumatic venous sinus thrombosis?
   1. Yes
   2. No
   3. Maybe
   4. N/A
