# Supplementary material for: Management of Traumatic Cerebral Venous Sinus Thrombosis: A United Kingdom and Ireland Survey on Practice Variation
Source: Neurotrauma Rep. 2024 Jun 6;5(1):540–51. doi: 10.1089/neur.2023.0118 (PMC11285999; doi:10.1089/neur.2023.0118)
Supplement: Supplementary Data S2 [file neur.2023.0118_supplementarymaterial_2.docx]

**Supplementary Material 2: free text responses to survey Q6:**

**‘If you give anticoagulation in selected cases, what are your criteria?’**

- *Dependent on extent of bleeding, GCS, ICP, progression on serial scans*
- *Progressive or obstructive venous sinus thrombosis or uncontrolled raised ICP. Lack of other contraindications such as co-existing coagulopathy.*
- *Is sinus occluded, likelihood of needing surgical intervention, co-morbidities and risk factors*
- *after weighing up the risk and benefits considering the TBI*
- *symptomatic*
- *Anticoagulate where possible, balanced against perceived risk of bleeding associated with other coexisting injuries and anticipated surgical procedures*
- *level of injury, time from injury, surgical intervention, other risk factors for bleeding*
- *depends on blood volume, ICP load, extent of thrombus,*
- *OCCLUSIVE THROMBUS, INTRA-CRANIAL HYPERTENSION, SECONDARY VENOUS INFARCT*
- *If no large parenchymal contusions, would anticoagulant. If large contusions with high risk of further blossoming, might wait 48h.*
- *Occlusive thrombosis, large, no evidence active bleeding, able to monitor (clinically or via ICP)*
- *On specific haematology advice, if no contusions or traumatic bleed that would contraindicate anticoagulation, if radiological evidence of venous infarct*
- *Liason with neurology on case by case basis*
- *thrombus within sinus with swelling +/- haemorrhage within the area drained by the vein in question*
- *Cerebral hypodensity with no intracerebral contusions/haematoma.*
- *We usually anticoagulate with advice from Stroke neurologist. Occasionally the severity of TBI may preclude*
- *Balance of ICp effect from contusions or CVST*
- *Risk/benefit assessment: Extent of thrombosis, evidence of venous hypertension, extent of other contusions/brain injury*
- *Absence of parenchymal contusions / ICH.*
- *Occlusive/ partly occlusive*
- *No fixed criteria. Age, extent of thrombosis. Likely need for a procedure. Often involve discussion with neurology and/or haematology*
- *Absence of progressive haemorrhagic contusion or unevacuated SOL,*
- *No other intracranial bleed, repeat CT at 48 hrs showing no evolving contusions, Dominant sinus involved.*
- *as per hematologist*
- *refractory raised ICP secondary to occlusive VST with no surgical haematomas driving ICP*
- *Confirmed CVST on CT Venogram and if patient is symptomatic clinically*
- *discuss with haematology. size of CVST. propagation/asleep patient cannot be monitored in terms of symptoms.*
- *Large occlusive symptomatic VST*
- *Extent of contusions / traumatic SAH, risk of bleeding (intracranial and other injuries), VTE risk*
